# Cdkl5 mutant zebrafish shows skeletal and neuronal alterations mimicking human CDKL5 deficiency disorder

**DOI:** 10.1038/s41598-022-13364-1

**Published:** 2022-06-04

**Authors:** Tatiana Varela, Débora Varela, Gil Martins, Natércia Conceição, M. Leonor Cancela

**Affiliations:** 1 Centre of Marine Sciences, University of Algarve, Faro, Portugal; 2 Faculty of Medicine and Biomedical Sciences, University of Algarve, Faro, Portugal; 3 Algarve Biomedical Center, University of Algarve, Faro, Portugal

**Keywords:** Genetics, Molecular biology, Diseases

## Abstract

CDKL5 deficiency disorder (CDD) is a rare neurodevelopmental condition characterized primarily by seizures and impairment of cognitive and motor skills. Additional phenotypes include microcephaly, dysmorphic facial features, and scoliosis. Mutations in cyclin-dependent kinase-like 5 (*CDKL5*) gene, encoding a kinase essential for normal brain development and function, are responsible for CDD. Zebrafish is an accepted biomedical model for the study of several genetic diseases and has many advantages over other models. Therefore, this work aimed to characterize the phenotypic, behavioral, and molecular consequences of the Cdkl5 protein disruption in a *cdkl5* mutant zebrafish line (sa21938). *cdkl5*^*sa21938*^ mutants displayed a reduced head size, suggesting microcephaly, a feature frequently observed in CDD individuals. Double staining revealed shorter craniofacial cartilage structures and decrease bone mineralization in *cdkl5* homozygous zebrafish indicating an abnormal craniofacial cartilage development and impaired skeletal development. Motor behavior analysis showed that *cdkl5*^*sa21938*^ embryos had less frequency of double coiling suggesting impaired glutamatergic neurotransmission. Locomotor behavior analysis revealed that homozygous embryos swim shorter distances, indicative of impaired motor activity which is one of the main traits of CCD. Although no apparent spontaneous seizures were observed in these models, upon treatment with pentylenetetrazole, seizure behavior and an increase in the distance travelled were observed. Quantitative PCR showed that neuronal markers, including glutamatergic genes were dysregulated in *cdkl5*^*sa21938*^ mutant embryos. In conclusion, homozygous *cdkl5*^*sa21938*^ zebrafish mimic several characteristics of CDD, thus validating them as a suitable animal model to better understand the physiopathology of this disorder.

## Introduction

CDKL5 deficiency disorder (CDD) is a rare X-linked neurodevelopmental condition characterized primarily by early-onset seizures, which generally begin in the first months of life and impairment of cognitive and motor skills (e.g., stereotypical hand movements, motor rigidity and deficient language acquisition)^1,2^. However, a variety of additional features have also been observed including microcephaly, scoliosis, bruxism, and distinctive facial features^1,2^. Despite the severity of the disease, the mechanisms responsible for its onset remain unclear.

The disorder is associated with a loss of cyclin-dependent kinase-like 5 (CDKL5) function due to a variety of mutations, but the link between the type or location of mutations and symptoms is not clearly understood^3^. Human CDLK5 is encoded by a single-copy gene located on the X chromosome and although the disorder is essentially observed in heterozygous females due to dominant X-linked mutations, the most severe phenotypes are observed in males^2^. CDKL5 is a ubiquitous protein mainly expressed in the brain, testes and thymus and found both in the cytoplasm and in the nucleus, where it co-localizes with nuclear speckles^4^. It has a kinase catalytic activity responsible for its autophosphorylation as well as the phosphorylation of amphiphysin 1, its first identified substrate^5^. CDKL5 can also mediate MeCP2 phosphorylation, suggesting a molecular link between CDKL5 disorder and MeCP2-associated Rett syndrome^6^. More recently, CDKL5 was shown to interact with Shootin1 and contribute to the regulation of neuronal polarization^7^.

Although mammalian models suggest a potential for reversibility, there are no approved therapies yet and standard treatments do not provide substantial symptom relief. The development of alternative and/or complementary model organisms that may accelerate the development of therapeutic approaches should therefore be contemplated to gain valuable insights in this field. The fact that the existing mouse models for *Cdkl5* knockout (KO) do not completely reproduce the human CDKL5 phenotype makes the use of alternative models such as zebrafish mutant lines even more interesting. Indeed, as referred in a recent review, despite all the advantages of rodent models, none of the available mutant *Cdkl5* mouse models develop early-onset seizures, a central feature of this syndrome in humans^8^. In contrast, it was recently shown that zebrafish, upon treatment with pentylenetetrazole (PTZ) can develop seizures, allowing researchers to investigate the molecular basis of this phenotype^9^.

Zebrafish is a suitable biomedical model that has been established as a genetically manipulable system^10^ and shares many physiological processes with humans including a large structural and functional conservation between zebrafish and human genes^11^. Zebrafish has a single *cdkl5* gene and two alternative splice variants, similar to human *CDKL5*, were recently identified. Variant 1 is mainly expressed in the brain, while variant 2 is ubiquitously expressed^12^. Although the function and mode of action of Cdkl5 in fish have not been investigated, previous studies conducted in our laboratory showed that *cdkl5* expression in zebrafish is in accordance with its known localization in humans^13^. Also, zebrafish *cdkl5* structural organization, flanking genomic regions, and putative transcription factor binding sites located in its promoter region appear to be highly conserved when compared to its human ortholog^13^. The corresponding proteins also show a high degree of sequence conservation, particularly in the catalytic domains required for phosphorylation^13^. These data support the suitability of zebrafish as a valid model to investigate CDKL5 deficiency disorder.

The main objective of this study was to characterize a stable zebrafish model (*cdkl5*^*sa21938*^) expressing a mutant form of Cdkl5, in terms of their morphological and behavior phenotype as well as their molecular alterations. The overall goal is to provide a new model organism to further investigate the Cdkl5 signaling pathway and mechanisms that lead to CDKL5 deficiency disorder and ultimately, contribute to the identification of therapeutic targets to treat or relief symptoms of CDD.

## Results

### Characterization of *cdkl5* mutation present in the sa21938 stable zebrafish line

CDKL5 loss-of-function in humans is known to be responsible for the CDKL5 deficiency disorder phenotype. To investigate the effect of Cdkl5 loss-of-function, a mutant *cdkl5* zebrafish line (sa21938) generated through the Zebrafish Mutation Project by ENU mutagenesis of males was characterized in this work.

**Figure 1:**
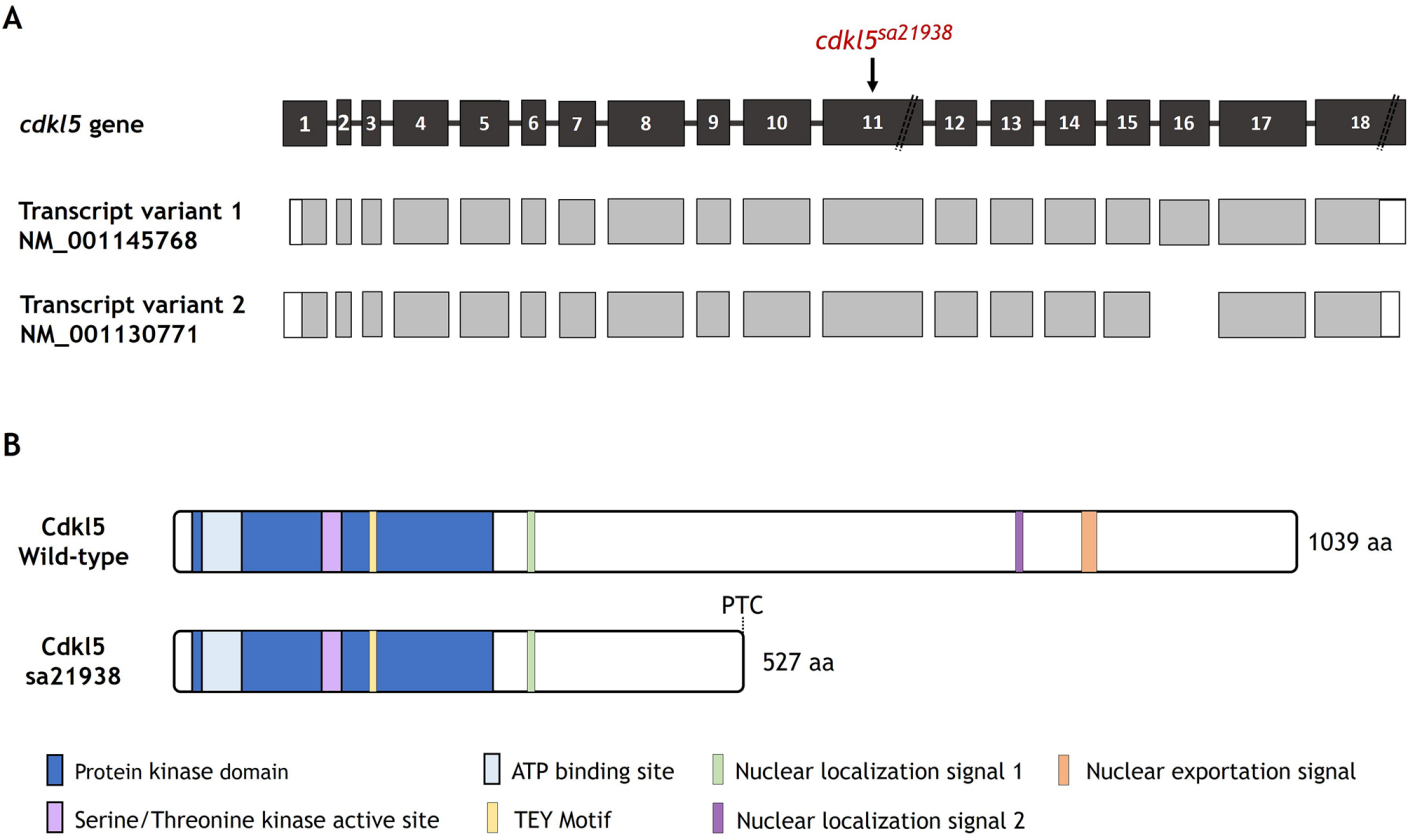
Characterization of *cdkl5*^*sa21938*^ mutation. (**A**) Localization of sa21938 mutation in zebrafish *cdkl5* gene. Exons and introns are represented by boxes and solid lines, respectively. In the structure of transcripts, white and grey boxes represent untranslated regions and coding regions, respectively. *cdkl5* gene (ENSDARG00000015240) and transcripts (NM_001145768 and NM_001130771) were retrieved from NCBI and Ensembl databases, respectively. Exons are in scale except exons 11 and 18. (**B**) Scheme of the wild-type and mutant Cdkl5 protein. PTC indicates a premature termination codon. Cdkl5 protein (NP_001124243) is available in NCBI database.

Zebrafish *cdkl5* has two splice variants encoding two proteins with 1039 and 1080 amino acids (aa), consisting of an N-terminal catalytic domain and a C-terminal region containing two nuclear localization signals and one nuclear export signal. *cdkl5*^*sa21938*^ mutants have a nonsense mutation in exon 11 of the *cdkl5* gene (Fig. 1A) consisting of an alteration of cytosine by an adenine (Chr 11: 30,219,227 C > A) leading to the introduction of a premature termination codon. As a consequence, the mRNA could be susceptible to degradation by the nonsense-mediated mRNA decay (NMD) mechanism or result in the production of a truncated protein with 527 aa, lacking one of the nuclear localization signals and the nuclear exportation signal located in the C-terminus (Fig. 1B). If produced, the truncated protein is likely mislocalized in the cell.

### Morphological characterization of *cdkl5*^*sa21938*^ mutant zebrafish

**Figure 2:**
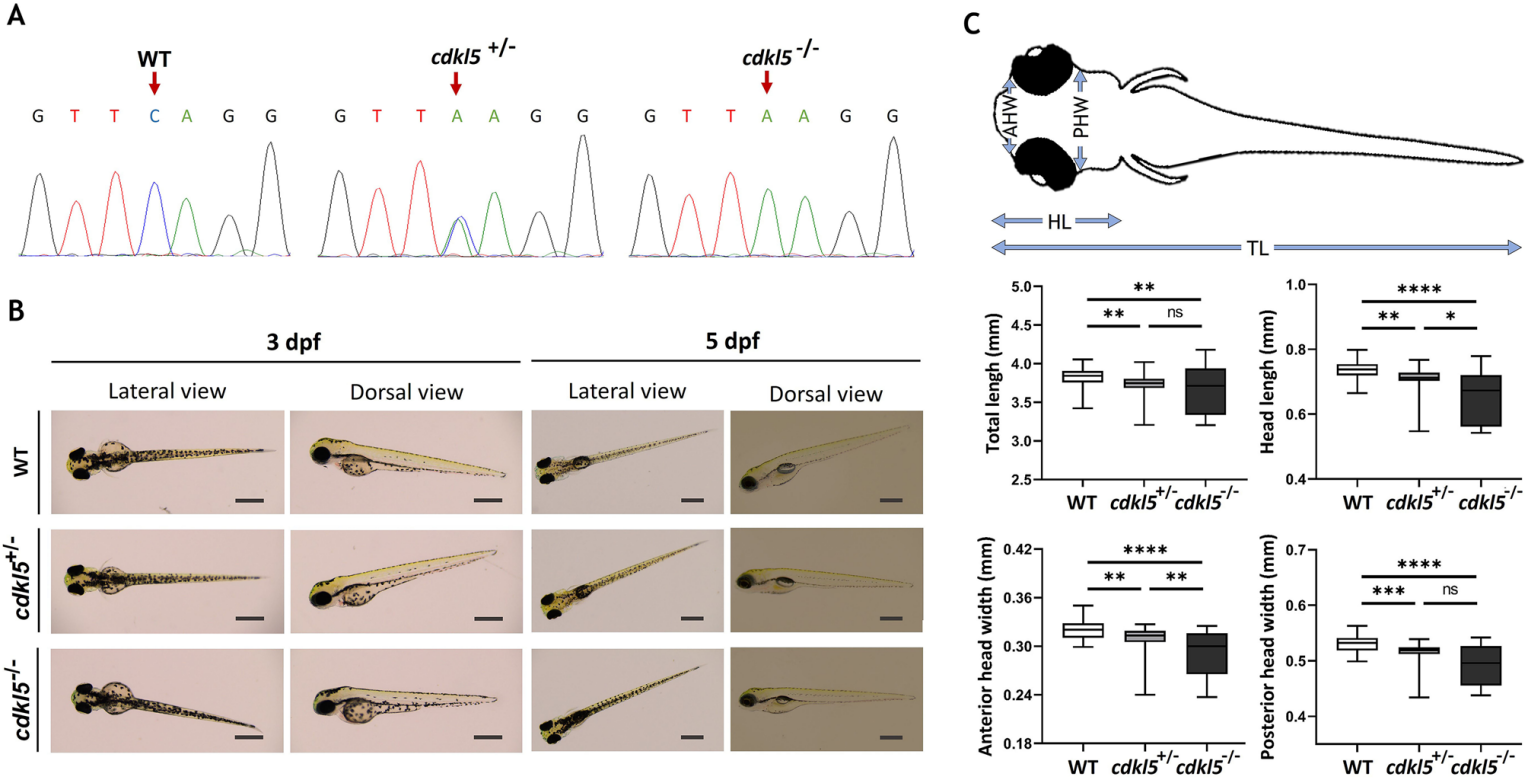
Characterization of *cdkl5*^*sa21938*^ mutant at initial larval stages. (**A**) Sequencing chromatograms showing the zebrafish genotypes for *cdkl5*. Arrows indicate the mutation site. (**B**) Representative lateral and dorsal images of wild-type (WT), heterozygous (*cdkl5*^+*/*−^), and homozygous (*cdkl5*^−^/^−^) mutant embryos with 3 dpf and 5 dpf. Scale bar = 0.5 mm. (**C**) Morphometric analysis of 5 dpf WT (n = 51), cdkl5^+/−^ (n = 50) and cdkl5^−/−^ (n = 71) embryos. Data are presented as median with interquartile range. Three independent experiments were performed. Statistical analysis was performed using Kruskal–Wallis followed by Dunn multiple comparisons test. *, **, ***, **** indicate *p* < 0.05, *p* < 0.01, *p* < 0.001 and *p* < 0.0001, respectively. ns indicates not significant. TL-total length; HL-head length; AHW-anterior head width; PHW-posterior head width.

The zebrafish obtained from EZRC European Zebrafish Resource Center (EZRC; www.ezrc.kit.edu) were genotyped to find heterozygous *cdkl5*^*sa21938*^ mutants and then in-crossed to obtain homozygous zebrafish. Sequencing results confirmed the existence of the three possible resulting genotypes (Fig. 2A). It was not possible to confirm the decrease of Cdkl5 protein levels by western blotting in these mutants due to the lack of specificity of the human anti-CDKL5 antibody to recognize the protein in zebrafish.

The phenotype regarding morphological features of the heterozygous (*cdkl5*^+/−^) and homozygous (*cdkl5*^−/−^) mutants were characterized throughout the first days of development. No severe morphological defects were observed in the mutants compared to wild-type embryos during the initial stages of development (Fig. 2B). Several morphometric parameters were analyzed in 5 dpf mutant and wild-type embryos (Fig. 2C). *cdkl5* mutant embryos have a smaller total body length compared to wild-type embryos. Both heterozygous and homozygous embryos display a significantly reduced head length, anterior head width and posterior head width than wild-type embryos, being more prominent in the cdkl5^−/−^ embryos.

**Figure 3:**
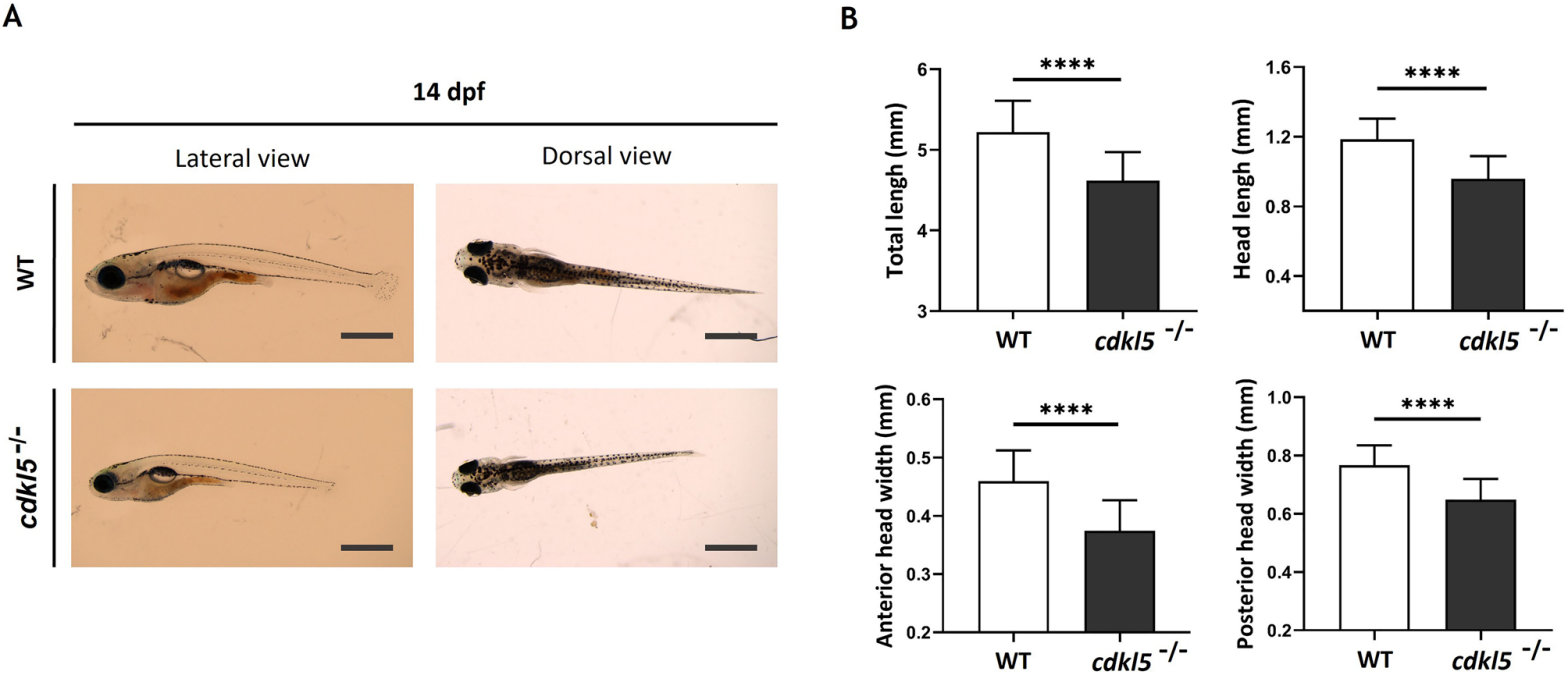
Morphological analysis of *cdkl5*^*sa21938*^ mutant at 14 dpf. (**A**) Representative lateral and dorsal images of wild-type (WT) and homozygous (*cdkl5*^−/−^) mutant larvae. Scale bar = 1 mm. (**B**) Morphometric analysis of WT (n = 30) and cdkl5^−/−^ (n = 25) larvae. Data are presented as mean ± SD. Three independent experiments were performed. Statistical analysis was performed using t-test with Welch’s correction. **** indicate *p* < 0.0001.

Morphological characterization of *cdkl5* homozygous mutant was also evaluated at an older stage of development (Fig. 3A). Similarly, homozygous mutant larvae at 14 dpf continued to show smaller total body length and displayed reduced head length, anterior head width and posterior head width compared to wild-type larvae (Fig. 3B). These results suggest a microcephaly phenotype, similarly to a known characteristic of CDKL5 deficiency disorder in humans^14^.

### Skeletal development of *cdkl5*^*sa21938*^ mutant zebrafish

**Figure 4:**
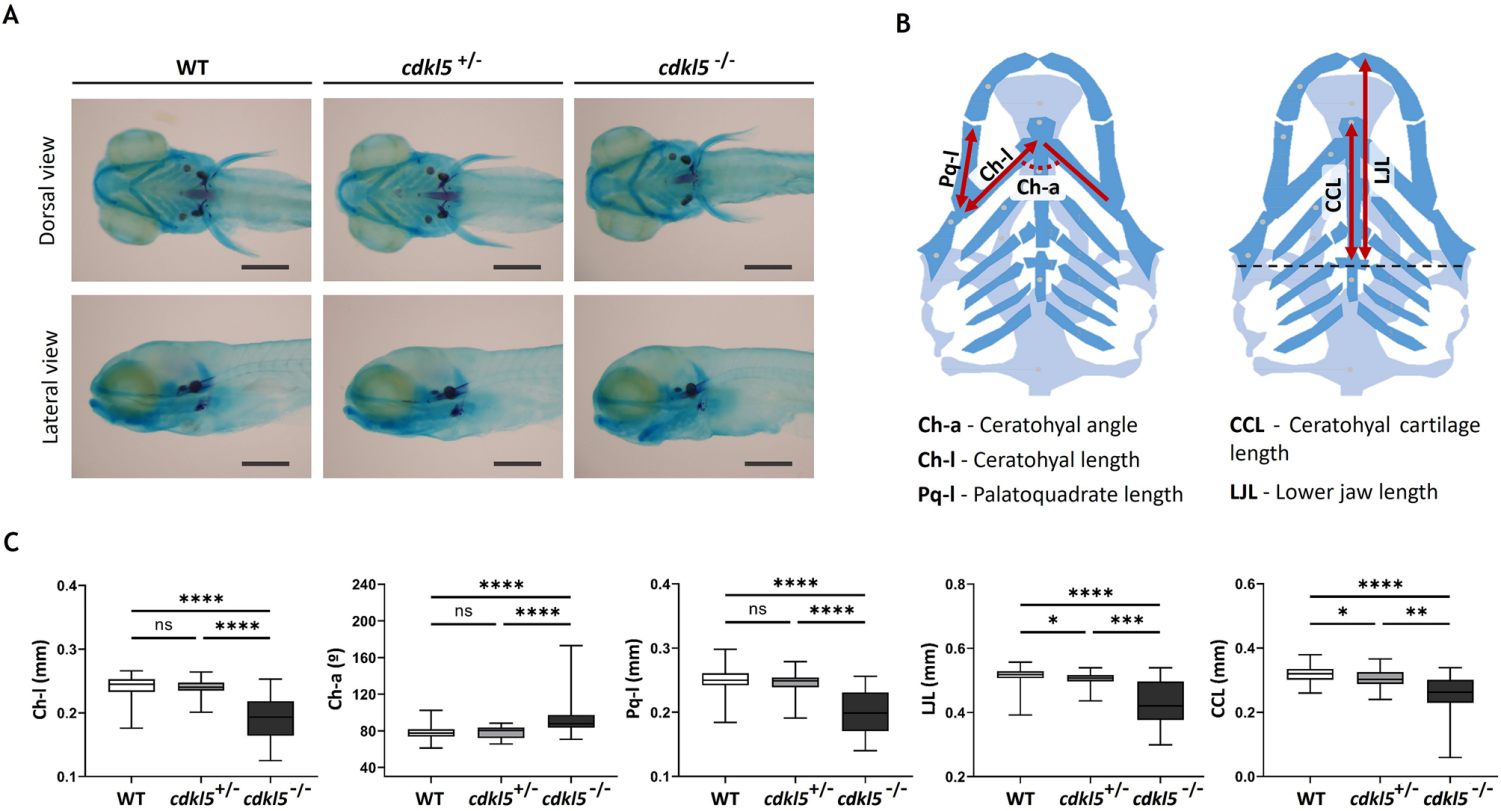
Craniofacial development of *cdkl5*^*sa21938*^ mutants with 5 dpf. (**A**) Representative images of the wild-type (WT), heterozygous (*cdkl5*^+/−^), and homozygous (*cdkl5*^−/−^) embryos double-stained with alcian blue and alizarin red. Scale bar = 0.3 mm. (**B**) Illustration of the analyzed craniofacial cartilage parameters. Adapted from Raterman et al.^36^ (**C**) Measurements of craniofacial cartilage parameters of WT (n = 59), *cdkl5*^+/−^ (n = 53) and cdkl5^−/−^ (n = 30) embryos. Three independent experiments were performed. Values are represented as median with interquartile range. Statistical analysis was performed using Kruskal–Wallis followed by Dunn multiple comparisons test. *, **, *** and **** indicate *p* < 0.05, *p* < 0.01, *p* < 0.001 and *p* < 0.0001, respectively. ns indicates not significant.

CDKL5 has been suggested to play a role in bone metabolism^2,15^. To investigate the consequence of Cdkl5 loss-of-function in craniofacial and axial skeleton development, *cdkl5*^*sa21938*^ mutant and WT zebrafish with 5 dpf and 14 dpf were double-stained with alcian blue to visualize the cartilage and alizarin red to visualize calcified bone structures. At 5 dpf, all craniofacial cartilage structures were visible and there were no evident morphology differences between WT and *cdkl5* heterozygous mutant embryos. However, homozygous mutant embryos displayed an altered craniofacial morphology (Fig. 4A). The craniofacial cartilage structures were evaluated by measuring the ceratohyal angle (Ch-a), ceratohyal length (Ch-l), palatoquadrate length (Pq-l), ceratohyal cartilage length (CCL), and lower jaw length (LJL) (Fig. 4B). Both heterozygous and homozygous mutant embryos displayed a significantly reduced CCL and LJL, being more pronounced in the *cdkl5*^−/−^ embryos. Additionally, *cdkl5*^−/−^ embryos also showed a significantly shorter Ch-l and Pq-l compared to *cdkl5*^+/−^ and WT embryos (Fig. 4C). The ceratohyal angle was significantly increased in the *cdkl5*^−/−^ embryos. Mineralized bone formation stained in red showed no abnormalities in 5 dpf *cdkl5* mutants compared to WT embryos. These data suggest that *cdkl5* homozygous mutant zebrafish present an abnormal cartilage development.

**Figure 5:**
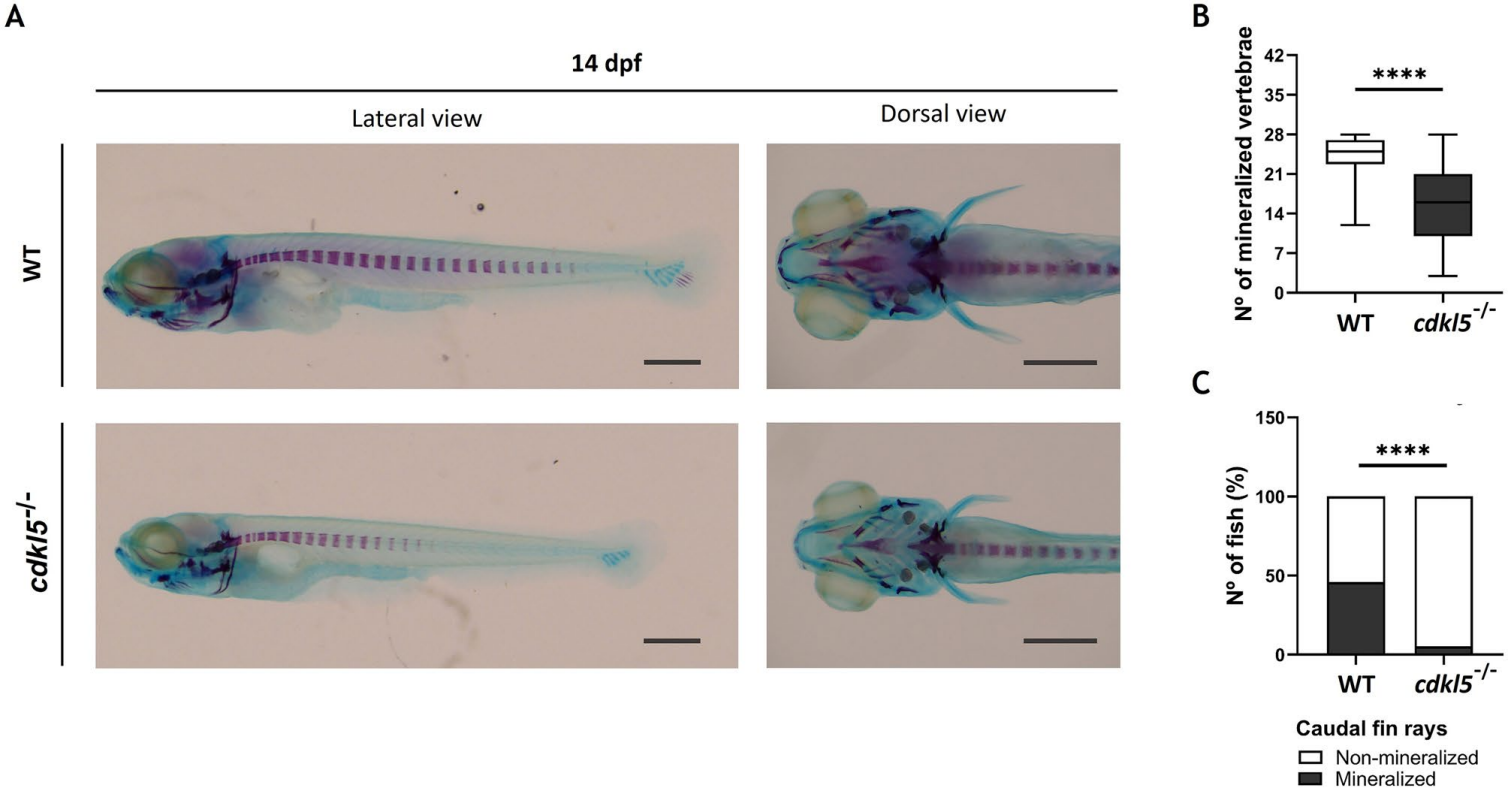
Skeletal development of *cdkl5*^*sa21938*^ mutants with 14 dpf. (**A**) Representative images of the wild-type (WT) and homozygous (*cdkl5*^-/-^) larvae double-stained with alcian blue and alizarin red. Scale bar = 0.5 mm. (**B**) Number of mineralized vertebrae in *cdkl5*^−/−^ (n = 58) and WT (n = 54) larvae. Values are represented as median with interquartile range. Statistical analysis was performed using Mann–Whitney. (**C**) Percentage of fish with mineralized caudal fin rays. Statistical analysis was performed using the Chi-square test. **** indicate *p* < 0.0001.

At 14 dpf, our results showed that the level of mineralized areas in homozygous *cdkl5* mutants stained with alizarin red was decreased compared to the WT larvae (Fig. 5A). During this stage of development, the number of mineralized vertebrae in *cdkl5*^−/−^ larvae was significantly lower than WT (Fig. 5B) and mainly found in the anterior part of the axial skeleton. The mineralized caudal fin rays were also significantly lower in *cdkl5*^−/−^ compared to the WT larvae, with only 5% of the homozygous mutant larvae presenting mineralized rays in the caudal fin (Fig. 5C). These results suggest an impairment or delay of bone mineralization in *cdkl5* mutants.

### Motor behavior of *cdkl5*^*sa21938*^ mutant zebrafish

One of the main characteristics of CDKL5 deficiency disorder is the impairment of motor skills, such as the limited ability to walk. Therefore, we analyzed the locomotor behavior of *cdkl5*^*sa21938*^ mutant embryos.

**Figure 6:**
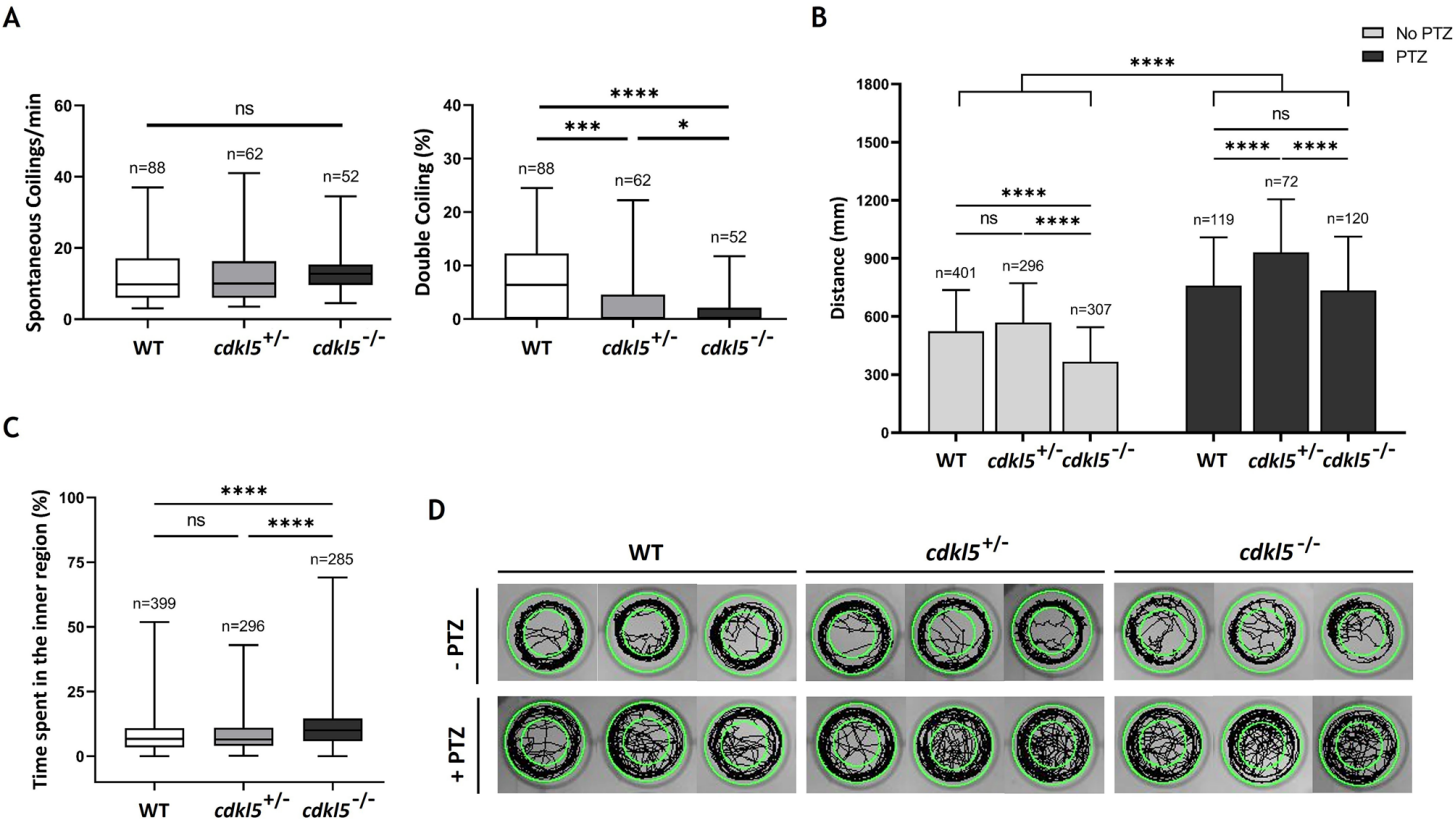
Behavior phenotype of mutant *cdkl5* zebrafish (sa21938 line). (**A**) Spontaneous coiling and percentage of double coiling of wild-type (WT), heterozygous (*cdkl5*^+/−^) and homozygous (*cdkl5*^−/−^) embryos at 24-hpf. Data are presented as median ± interquartile range. (**B**) Total distance travelled by *cdkl5*^+/−^ and *cdkl5*^−/−^ mutant zebrafish embryos and WT embryos with 5 dpf, treated or not with PTZ. Data are presented as mean ± SD. (**C**) Percentage of time spent in the inner region of the well by 5 dpf WT, *cdkl5*^+/−^ and *cdkl5*^−/−^ embryos. Data are presented as median with interquartile range. Statistical analysis was performed using one-way ANOVA followed by Tukey’s test (**B**) and Kruskal–Wallis followed by Dunn multiple comparisons test (**A** and **C**). *, *** and **** indicate *p* < 0.05, *p* < 0.001 and *p* < 0.0001, respectively. ns indicates not significant. (**D**) Representative images of the locomotive trajectory of 5 dpf WT, *cdkl5*^+/−^ and *cdkl5*^−/−^ embryos.

The first motor activity of embryonic zebrafish is the spontaneous coiling which can be classified into two groups: single coiling and double coiling. Single coiling consists of a single contraction of the trunk, after which the tail returned to its resting position. While double coiling consists of two contralateral contractions of the trunk within 1 s. Between these two contractions, the tail does not return to a resting position^16^. Spontaneous contractions of 25 hpf dechorionated embryos were monitored. Our results showed no significant differences in the number of spontaneous coiling per minute between the *cdkl5*^+/−^, *cdkl5*^−/−^ and wild-type embryos. However, mutant embryos presented less percentage of double coiling compared to the wild-type embryos, being this decrease more pronounced in the homozygous embryos (Fig. 6A). The arising of double coiling represents an event in the stepwise maturation from a single coiling behavior to swimming. While single coiling is independent of chemical neurotransmission, double coiling is dependent on both electrical and glutamatergic transmission^16^. Therefore, results suggest that the glutamatergic transmission of *cdkl5*^*sa21938*^ mutants may be impaired or delayed.

The spontaneous swimming behavior of mutant and wild-type embryos at 5 dpf was also monitored for 5 min in a 24-well plate. The distance travelled by the *cdkl5*^−/−^ mutant embryos was significantly decreased compared to the *cdkl5*^+/−^ and wild-type embryos, suggesting an impaired locomotor activity (Fig. 6B). The thigmotaxis i.e., the tendency to avoid the center of an arena and swim close to the borders of the well, was also analyzed. *cdkl5*^−/−^ embryos spent slightly but significantly more time in the inner region of the well compared to the *cdkl5*^+/−^ and WT embryos (Fig. 6C), suggesting a reduced thigmotactic behavior.

To investigate the response to PTZ, a seizure-inducing drug that is a GABA receptor inhibitor^17^, WT and *cdkl5*^*sa21938*^ mutant embryos at 5 dpf were exposed to 16 mM of PTZ. A seizure-like behavior such as rush, circular swimming motion followed by a series of brief clonus-like convulsions^18^ was observed in WT, *cdkl5*^+/−^ and *cdkl5*^−/−^ embryos. The three groups of embryos also presented a significant increase in the distance travelled. However, that increase was significantly greater in the homozygous mutants compared to the wild-type fish thus indicating higher susceptibility to seizures. The spontaneous and PTZ induced locomotor trajectories of WT, *cdkl5*^+/−^ and *cdkl5*^−/−^ embryos are represented in Fig. 6D.

### Molecular analysis of *cdkl5*^*sa21938*^ mutant zebrafish

**Figure 7:**
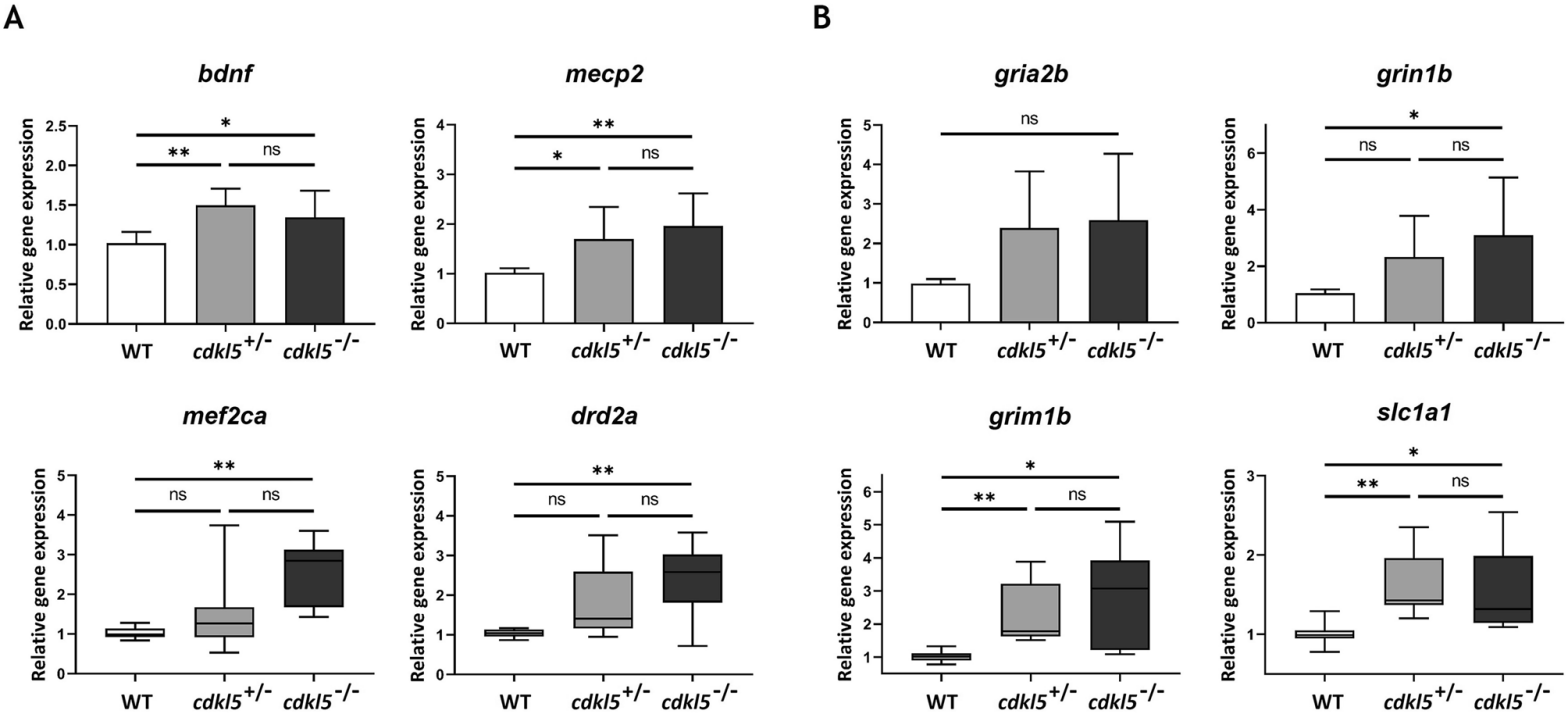
Molecular analysis of *cdkl5*^*sa21938*^ mutants. Relative gene expression analysis of neuronal markers (**A**), including glutamatergic neurotransmission markers (**B**) in wild-type (WT), heterozygous (*cdkl5*^+/−^), and homozygous (*cdkl5*^−/−^) mutant zebrafish embryos at 5 dpf, measured by qPCR. For *bdnf*, *mecp2*, *gria2b* and *grin1b*, values are presented as mean ± SD and statistical analysis was performed using one-way ANOVA followed by Tukey’s test. For *mef2ca*, *drd2a*, *grim1b* and *slc1a1*, data are presented as median with interquartile range and statistical analysis was performed using Kruskal–Wallis followed by Dunn multiple comparisons test. At least three independent experiments were performed. *, and ** indicate *p* < 0.05 and *p* < 0.01, respectively. ns indicates not significant.

To investigate the molecular pathways affected by a loss of Cdkl5 function in zebrafish, gene expression analysis of several neuronal associated genes known to be related to CDKL5 or Rett syndrome was performed by qPCR in WT, *cdkl5*^+/−^ and *cdkl5*^−/−^ mutant embryos at 5 dpf. Our results showed alterations in the expression of the analyzed neuronal markers. The methyl CpG binding protein 2 (MECP2), associated with Rett syndrome, is a chromatin-associated protein that binds to methylated CpG sites of genes, regulating their activity^19^. It was demonstrated that MECP2 represses *CDKL5* transcription and that CDKL5 can phosphorylate MECP2^20^. MECP2 is also known to affect the activity of *BDNF* (Brain-derived neurotrophic factor) which is an important regulator of neuronal development and function^21,22^. Interestingly, studies showed that CDKL5 regulates neuronal morphogenesis through a mechanism involving BDNF-Rac1 signaling^6^. In our study, levels of *bdnf* and *mecp2* were equally significantly increased in *cdkl5* heterozygous and homozygous mutant embryos compared to the WT embryos (Fig. 7A), indicating dysregulation of these pathways due to Cdkl5 loss-of-function. BDNF was shown to activate several members of the myocyte enhancer factor 2 (MEF2) family of transcription factors^23^. MEF2C mutations cause severe mental retardation with phenotypical overlap to Rett syndrome and can significantly reduce *MECP2* and *CDKL5* expression^24^. The expression of Dopamine Receptor D2 (*Drd2*), a major mediator of dopamine effects with diverse functions such as cognition, movement, learning and memory, was found increased in the striatum of *Mecp2* mutant mice^25^. Our results showed that levels of *mef2ca* and *drd2a* were also significantly increased but only in the homozygous embryos (Fig. 7A).

Since impairment of the glutamatergic neurotransmission in *cdkl5*^*sa21938*^ mutants was suggested by our spontaneous motor behavior results, the expression of glutamatergic genes was also evaluated. We analyzed the expression of different types of glutamate receptors, namely the ionotropic NMDA receptor *grin1b*, the ionotropic AMPA receptor *grin2a*, the metabotropic receptor *grm1b,* and the glutamate transporter *slc1a1*. Levels of *grin1b* were significantly increased in the *cdkl5*^−/−^ mutant embryos compared to WT embryos. Levels of *grm1b*, and *slc1a1* were increased in *cdkl5*^+/−^ and *cdkl5*^−/−^ mutant embryos (Fig. 7B). Although an increase in the expression of *gria2b* was observed, no statistically significant differences were found between WT and *cdkl5*^*sa21938*^ mutant embryos (Fig. 7B).

## Discussion

CDKL5 deficiency disorder is a drug-resistant epileptic encephalopathy and currently no approved therapies are available for its treatment^26^. Several mouse models have been developed to gain insights into the molecular changes underlying CDD and to develop new treatments^27–30^. Zebrafish is a suitable biomedical model and shares many physiological processes with humans^11^. Additionally, it offers important advantages over other models that could be relevant to study CDD, such as translucent embryos and larvae, allowing the visualization of organs during early development, and easy access for genetic manipulation. Furthermore, it is also well suited to high-throughput drug screening^31–34^. In our study, we characterized the morphology, motor behavior, and molecular changes of the *cdkl5*^*sa21938*^ zebrafish mutant line at its initial developmental stages. ENU-mutagenesis can originate other mutations along with the desired mutation. To exclude the possible effect of undesired mutations our experiments were performed using fish generated from several outcrosses.

*cdkl5*^*sa21938*^ mutant zebrafish did not show an obvious phenotype, however detailed morphological analysis revealed a reduced head size already apparent in heterozygous but more pronounced in homozygous mutants in comparison with the wild-type. Our results suggest that Cdkl5 loss-of-function leads to a microcephaly phenotype in zebrafish, a feature frequently observed in patients with CDKL5 deficiency disorder^14^. Our results are in agreement with the recently published data by Serrano et al. who also showed the presence of a microcephaly phenotype in this mutant^35^. Further studies could be performed to confirm this observation, such as sections of the head to visualize brain size.

CDKL5 has been suggested to have a potential role in bone metabolism. A recent study showed that it was hypermethylated in all the osteoporotic participants^15^. Furthermore, scoliosis and several dysmorphic facial features such as midface hypoplasia, high forehead, large and deep-set eyes, full lips, high palate, and widely spaced teeth have been identified in individuals diagnosed with CDD^2^. Since zebrafish is considered a robust model for the craniofacial abnormalities observed in humans^36^, we investigated the Cdkl5 loss-of-function effect in embryos and larvae craniofacial and skeletal development. The *cdkl5* homozygous mutants presented mild cartilage development defects, such as hypoplasia of palatoquadrates and ceratohyals cartilages and wider ceratohyal angle, which may correlate with the phenotype observed in individuals with CDD. To shed light on the observed changes, the expression levels of genes related to cartilage should be performed. Accordingly, a significant decrease in the ceratohyal cartilage distance was also described by Serrano et al. However, they observed a decrease in the ceratohyal angle^35^, contrary to what was observed in our study. This discrepancy could be due to the low number of fish analyzed (n = 13) in their study. In our study, an overall decrease in bone mineralization was observed in homozygous *cdkl5* mutants in comparison to wild-type larvae at 14 dpf. Altogether, these results indicate that Cdkl5 is needed for both normal cartilage craniofacial development and skeletal development. The presence of skeletal deformities in adult mutant fish should be evaluated to further elucidate the Cdkl5 role in bone formation.

Motor dysfunction is a prominent feature of CDKL5 deficiency disorder. Since zebrafish is recognized as a valuable model to study genetically linked motor defects and behaviors observed in neurodevelopmental human diseases^37^, the motor behavior of *cdkl5*^*sa21938*^ mutants was investigated. No differences were observed in the frequency of spontaneous single contractions, that is the earliest embryonic motor behavior, between *cdkl5*^*sa21938*^ mutants and wild-type embryos. Spontaneous coiling is driven by excitatory activity in the spinal cord and it is only dependent on the electrical coupling of the neuronal network. Subsequently, as chemical neurotransmission develops, mature locomotion is achieved^16^. Our results indicate that excitation in the spinal cord is not affected by Cdkl5 loss-of-function. The double coiling represents a transition behavior between single coiling and swimming activity driven by chemical glutamatergic and glycinergic neurotransmission^16^. *cdkl5*^*sa21938*^ embryos showed a reduced percentage of double coiling suggesting that Cdkl5 deficiency results in abnormal glutamatergic neurotransmission. Locomotor behavior analysis revealed that the swimming activity was reduced in *cdkl5* homozygous embryos, which covered a shorter swimming distance. Accordingly, a significant decrease in distance swum by the homozygous mutant was also reported by Serrano et al.^35^. The available works using *CDKL5* KO mice showed contradictory behavioral results among them, with both hypoactivity and hyperactivity reported in these studies^27–29,38^. Altogether, these results suggest that Cdkl5 ablation in zebrafish causes impairment in their motor activity, following what was observed in individuals with CCD that have impaired motor skills such as delayed or impaired walking.

The thigmotaxis behavior used to evaluate the level of anxiety was also investigated. Homozygous *cdkl5* embryos showed a slight but significant reduced thigmotaxis. These results may suggest that embryos with defective Cdkl5 have anxiety behavioral alterations or could be sensitive to the tactile stimuli represented by the wall of the well, a behavior that resembles the autistic features in CDD individuals. In accordance, Wang et al. reported a decreased anxiety-related behavior and autistic-like behavior in *Cdkl5* KO mice^30^.

Although early-onset seizures are a central characteristic of CDKL5 deficiency disorder, spontaneous seizures were not apparent in *cdkl5*^*sa21938*^ zebrafish embryos, at least through the analysis of the mutant fish swimming behavior with the tested conditions (5 min of tracking and 5 dpf zebrafish). Nevertheless, we cannot affirm that the *cdkl5*^*sa21938*^ mutant does not have spontaneous seizures. The absence of spontaneous seizures could be simply because of the parameters used, such as the age of the fish and the time of tracking used for the analysis. Thus, the behavior of mutants at older stages of development should be further analyzed, and the time of tracking could be increased. Electroencephalographic records should also be performed to investigate the epileptic phenotype. Interestingly, Serrano et al. identified the existence of spontaneous seizures in *cdkl5* zebrafish mutants through live imaging of the brain by using a transgenic line expressing a genetically-encoded calcium indicator. This represents an advantage over the existent *Cdkl5* mouse models that do not exhibit spontaneous seizures at initial developmental stages^27,29,30^. Nevertheless, our results showed that after treatment with a seizure-inducing drug, a seizure behavior was achieved by the homozygous fish, with an increase in their swimming activity. And that increase was significantly greater in the homozygous mutants compared to the wild-type fish thus, indicating higher susceptibility to seizures. This could be valuable to identify epileptic mechanisms affected by Cdkl5 loss-of-function in CDD. The latency, number of seizures, seizure times and the interval between seizures should also be investigated to better characterize the epileptic events.

To shed light on the molecular pathways affected by defective Cdkl5, the expression levels of brain-related genes were examined in the mutant *cdkl5*^*sa21938*^ embryos. The genes *bdnf, mecp2 mef2ca* and *drd2a* were upregulated in homozygous embryos. BDNF is crucial for the central nervous system development, and studies show that it is involved in epileptogenesis. Although there are contradictory data, some studies indicate that higher levels of BDNF induce epilepsy^39^. MECP2 is a methyl-CpG-binding protein important for appropriate neuronal development. In vivo studies showed that mice overexpressing MECP2 develop seizures and are less active^40^, therefore the presence of higher levels of this gene in mutant embryos provides further evidence for the presence of abnormal neuronal development/function. Since our results suggested an impairment of the glutamatergic neurotransmission in the *cdkl5*^*sa21938*^ mutants, we also analyzed the expression of glutamatergic-related genes, including the NMDA receptor *grin1b*, the AMPA receptor *grin2a*, the metabotropic receptor *grm1b*, and the glutamate transporter *slc1a1*. These genes were all overexpressed in the *cdkl5*^*sa21938*^ mutants, confirming that the glutamatergic pathway is affected. The connection between the dysregulation of expression of the analyzed genes and the observed phenotype should be further investigated, and the expression of additional genes should be analyzed to identify the molecular basis of the disease, for instance by performing RNA-seq.

In conclusion, our data show that homozygous *cdkl5*^*sa21938*^ zebrafish recapitulate a number of characteristics of CDKL5 deficiency disorder, such as the presence of microcephaly, craniofacial dysmorphic features, and locomotor behavior defects. Therefore, this work provides evidence that validates the use of this model organism to further study CDKL5 function and associated molecular pathways, in order to better understand the physiopathology of CDD. Furthermore, it represents a suitable in vivo model for the first-line screening of small-molecule libraries for total/partial rescue of phenotypes mimicking CDKL5 deficiency disorder.

## Methods

### Ethics statement

All procedures involving zebrafish experimentation were performed in accordance with the EU and Portuguese legislation for animal experimentation and welfare (Directives 86/609 CEE and 2010/63/EU; Decreto-Lei 113/2013; Portaria1005/92, 466/95 and 1131/97) and in compliance with ARRIVE guidelines (https://arriveguidelines.org). This study was approved by the Portuguese Direção-Geral de Alimentação e Veterinária (authorization no. 0421/2021). Animal handling and experimentation were performed by qualified operators. All efforts were made to minimize pain, distress, and discomfort. Experiments were terminated (fish were returned to normal conditions or euthanized) whenever adverse effects were observed.

### Fish maintenance

Mutant *cdkl5*^*sa21938*^ zebrafish line generated by Stemple lab^41^ was obtained from EZRC. Adult wild-type (WT) zebrafish (AB strain) and *cdkl5*^*sa21938*^ mutant zebrafish were maintained in a recirculating system at controlled room temperature (28 °C) and kept under a 14 h light -10 h dark cycle. For the reproductions, adult females were separated from males with plastic dividers in breeding boxes the night before spawning. The next day, they were allowed to spawn naturally. The embryos were raised in E3 medium with 0.1% methylene blue to avoid fungal contamination and staged according to the morphological scheme described by Kimmel et al.^42^. Dead or undeveloped embryos were removed daily. For the experiments using the *cdkl5*^*sa21938*^ mutant line, heterozygous embryos were obtained by crossing adults F3 homozygous mutants with AB wild-type zebrafish; homozygous embryos were obtained by incross adults F3 homozygous mutants, and AB wild-type zebrafish was used as control.

### Genotyping of *cdkl5*^*sa21938*^ zebrafish mutants

To extract genomic DNA (gDNA), adult zebrafish were anesthetized in tricaine methanesulfonate (MS222) solution (168 mg/L), and the tail fin was clipped and incubated at 95 °C in 25 μl alkaline lysis buffer containing 25 mM NaOH and 0.2 mM EDTA. After dissolution, 25 μl of neutralization buffer (40 mM Tris–HCl. pH = 8) was added. Then, the *cdkl5* fragment flanking the mutation was amplified by polymerase chain reaction (PCR) from 50 ng of gDNA using specific primers (Table 1) and Taq DNA polymerase (Invitrogen) following manufacturer instructions. PCR conditions were as follows: initial denaturation step at 94 °C for 3 min; 35 cycles of amplification (denaturation at 94 °C for 30 s; annealing at 57 °C for 30 s; extension at 72 °C for 15 s); and a final extension at 72 °C for 10 min. PCR products were separated in an agarose gel (1.2%) by electrophoresis, then visualized under ultraviolet light using Safe Green nucleic acid stain (Nzytech) and sequenced (CCMAR).

**Table 1:** List of primers used in this work.

Name	Sequence
***cdkl5* specific primers**	
*cdkl5*_mut_sa21938	Fw: ATGCCTTCCACGTCCTCCTC Rev: GTAGGCTTCGGTTCATCTGGT
**qPCR primers**	
*bdnf*	Fw: AGCTGAAGAGACAACTTGCAG Rev: CCATAGTAACGAACAGGATGGTC
*mecp2*	Fw: AGAGACCTTTGAGAAACGACTG Rev: TCTTCTTGTGACTCTTCGGTG
*mef2ca*	Fw: ATGAGCCTGAGCCGCAAAC Rev: TCCGCCCATCACTTCTCCA
*drd2a*	Fw: TGGTACTCCGGAAAAGACG Rev: ATCGGGATGGGTGCATTTC
*grin1b*	Fw: CTCCCTATTCCCACCAAGCCCA Rev: CTTTCTCTGCCTTTGTTTCCCTCTCC
*gria2b*	Fw: AGTATGGTGGAGCGAATGTGTCAGG Rev: TGAAGTGTACCGTATCTTGCTGTCTG
*grm1b*	Fw: ACACAGAGGGAAACTACGGTGAG Rev: CACTACACGAGCTTTGGGCAG
*slc1a1*	Fw: TCCTGATGAGAATGCTAAAGATGGT Rev: CATCACAAGAACGATTCCAAGAATGAC
*β-actin*	Fw: GATGCGGAAACTGGCAAAGG Rev: GAGGAGGGCAAAGTGGTAAACG

*Fw* Forward, *Rev* Reverse.

### Morphometric analysis of *cdkl5* mutant line

Phenotype of zebrafish *cdkl5* mutants was visualized and photographed at different developmental stages using a Leica MZ10F stereomicroscope. Parameters including body length, head length and head width were measured using ImageJ software.

### Alcian blue-Alizarin red double staining

Zebrafish with 5 dpf and 14 dpf were collected and sacrificed with a lethal dose of anesthesia (MS222). After washing twice with phosphate-buffered saline (PBS), zebrafish were fixed overnight in 4% paraformaldehyde (PFA; Sigma). They were washed twice with PBS and dehydrated in increasing ethanol series. Acid-free double staining was performed following Walker and Kimmel protocol^43^. Briefly, zebrafish were incubated with double stain solution (0.02% Alcian blue, 40 mM MgCl_2_, 0,005% Alizarin red S) overnight at room temperature under agitation. The pigmentation was removed by exposing the zebrafish to a bleaching solution of 1.5% H_2_O_2_ and 1% KOH for 20 min. Then, the tissue was cleared using successive changes of glycerol and KOH solution. Stained zebrafish were visualized and imaged using a stereomicroscope (Leica MZ10F). Several craniofacial parameters, such as ceratohyal angle (Ch-a), ceratohyal length (Ch-l), palatoquadrate length (Pq-l), ceratohyal cartilage length (CCL) and lower jaw length (LJL) were measured using Image J.

### Behavioral analysis

The motor behavior of *cdkl5* mutant zebrafish was characterized through several parameters and compared to wild-type or control fish. The rate of spontaneous coiling contractions was analyzed at 25 h post-fertilization (hpf). For that, embryos were dechorionated and placed in a 12-well plate filled with 2 ml of water. After 1 h of habituation each fish was recorded for 2 min using a stereomicroscope.

To monitor the spontaneous swimming activity, embryos with 5 days post-fertilization (dpf) were randomly placed in a 24-well plate filled in with 2 ml of water. Homogeneous illumination was provided, and swimming motor behavior was monitored for 5 min using the ZANTIKS apparatus. Parameters such as distance travelled, thigmotaxis and swimming patterns were analyzed. Embryos that did not swim were excluded from the analysis. To investigate thigmotactic behavior, wells were divided into two zones: an inner circular region (radius 4.5 mm) and an outer circular region (radius 8 mm); and the time spent by the embryos in each region was assessed.

Embryos’ behavior was also analyzed after treatment with PTZ, a seizure-inducing drug. For that, the water from each well was removed and 2 ml of PTZ solution (16 mM; Sigma) was added. After 10 min, swimming behavior was recorded for 5 min using the ZANTIKs apparatus.

### RNA extraction and cDNA synthesis

Pools of approximately 30 *cdkl5*^*sa21938*^ mutant embryos with 5 dpf were collected, sacrificed with a lethal concentration of anesthesia (MS22), and washed twice with PBS. Total RNA was extracted using NZYol (NZYTech) according to the manufacturer’s instructions and its quality was accessed through electrophoresis in an agarose gel stained with GreenSafe (NZYTech). At least three biological replicates were obtained from individual experiments. For reverse transcription, 1 µg of total RNA was subjected for 30 min at 37 °C to RQ1 RNase-free DNase (Promega) and cDNA was synthesized by the Moloney murine leukemia virus reverse transcriptase (Invitrogen) using an oligo-dT primer.

### Quantitative real-time PCR (qPCR)

Amplifications by qPCR were carried out in a total volume of 20 µl, containing 1 × NZYSpeedy qPCR Green Master Mix ROX plus (NZYTech), 0.4 μM of each forward and reverse gene-specific primers and 2 μl of cDNA (1:10 dilution). Gene-specific primers are listed in Table 1. qPCR reactions were performed in a CFX Connect Real-Time PCR Thermocycler (Bio-Rad) under the following conditions: an initial denaturation step at 95 °C for 2 min, then 40 cycles of amplification (each cycle is 5 s at 95 °C, 20 s at 60 °C). Relative gene expression was determined by 2^–∆∆Ct^ and normalized using β-actin as reference gene (Table 1). Two technical replicates for each sample were performed.

### Statistical analysis

Statistical analysis was performed using Prism 5 (GraphPad Software). Data normality was assessed using Kolmogorov–Smirnov test. Normally distributed data are presented as mean ± SD and significant differences were determined through one-way analysis of variance (ANOVA) followed by Tukey’s post-test for three groups and t-test with Welch correction for two groups. Data failing the normality test are presented as median with the interquartile region (boxplot) and significant differences were determined through Kruskal–Wallis followed by Dunn multiple comparisons test for three groups and Mann–Whitney test for two groups. The Chi-square test was used to analyze the occurrence of mineralized caudal fin rays. Differences were considered statistically significant for *p* < 0.05.

## Data Availability

The data generated and analyzed during this study are available from the corresponding author upon reasonable request.
